# Draft Genome Sequence of *Streptomyces bottropensis* ATCC 25435, a Bottromycin-Producing Actinomycete

**DOI:** 10.1128/genomeA.00019-13

**Published:** 2013-03-14

**Authors:** Hongyu Zhang, Wei Zhou, Yibin Zhuang, Xiaomei Liang, Tao Liu

**Affiliations:** a Key Laboratory of Systems Microbial Biotechnology, Tianjin Institute of Industrial Biotechnology, Chinese Academy of Sciences, Tianjin, China; b Tianjin University of Science and Technology, Tianjin, China

## Abstract

A series of bottromycin antibiotics have been isolated and identified from *Streptomyces bottropensis* strain ATCC 25435. Here, a draft genome sequence of *S. bottropensis* ATCC 25435 is presented. The genome carries an intact biosynthetic gene cluster for bottromycin antibiotics, which provides insight into the combinatorial biosynthesis of bottromycin antibiotics.

## GENOME ANNOUNCEMENT

*Streptomyces bottropensis* strain ATCC 25435 (DSM 40262), obtained from the Leibniz Institute DSMZ-German Collection of Microorganisms and Cell Cultures (DSMZ), has become an organism of interest due to its ability to produce bottromycin antibiotics (bottromycins A2, B2, and C2), which exhibited activities against methicillin-resistant *Staphylococcus aureus* (MRSA) and vancomycin-resistant enterococcus (VRE) (1–3). Herein, we report the draft genome sequence of *S. bottropensis* ATCC 25435. The whole genome sequence was determined by paired-end sequencing with high-throughput Illumina sequencing technology at the Beijing Genomics Institute (BGI) in China (4). Assembly was performed using the Short Oligonucleotide Alignment Program (SOAP)*denovo* 1.05 (5). Protein-encoding genes, rRNA operons, and tRNAs were predicted by Glimmer 3.0 (6), RNAmmer (7), and tRNAscan-SE (8), respectively. Functional annotation was based on BLASTp with the Kyoto Encyclopedia of Genes and Genomes (KEGG) (9), Swiss-Prot (10), Clusters of Orthologous Groups (COG) (11), and nonredundant (NR) databases.

All the reads up to 1,199 Mb were obtained, which represents a 134-fold coverage of the genome. Finally, we obtained the draft genome (G+C content, 71.53%) of *S. bottropensis* ATCC 25435, with a size of 8,914,727 bp distributed in 43 scaffolds that include 109 contigs.

The genome consists of one linear chromosome with 4 rRNA operons, 70 tRNA genes, and 8,253 protein-coding genes (CDSs); among the CDSs, 2,882 proteins could be assigned to COG families, 3,814 proteins could be assigned to KEGG orthology, and 734 proteins have no match to any proteins in the NR databases.

Genome analysis revealed a number of genes related to the biosynthesis of secondary metabolites. Twenty-one secondary-metabolite (4 siderophores, 5 terpenes, 1 lantibiotic, 1 bacteriocin, 2 polyketide synthase [PKS] I, 2 polyketide synthase II, 3 nonribosomal peptide synthetases [NRPS], and 3 hybrid NRPS-PKS) biosynthetic gene clusters were identified by antiSMASH (12). Many putative genes involved in antibiotic biosynthesis showed low identity with known ones, suggesting that *S. bottropensis* ATCC 25435 might be a producer of novel secondary metabolites.

The putative gene cluster for the biosynthesis of bottromycins, localized on the chromosome (scaffold 9), has a highly similar organization to the gene clusters described from *Streptomyces* sp. WMMB272 (13) and *Streptomyces* sp. BC 16019 (14). It contains all of the open reading frames (ORFs) reported in *Streptomyces* sp. BC 16019 and *Streptomyces* sp. WMMB272, including 3 different radical SAM-dependent enzyme genes, 1 precursor peptide gene, 2 cyclode hydratase (YcaO-like family) genes, 1 putative α/β hydrolase gene, 1 putative amidohydrolase gene, 1 cytochrome P450 enzyme gene, 1 transcriptional regulator gene, 1 *O*-methyltransferase gene, 1 multidrug transporter gene, and 1 leucyl-aminopeptidase gene. All of the putative proteins show extremely high similarities (most of them 99%) to their counterparts in *Streptomyces* sp. BC 16019 and *Streptomyces* sp. WMMB272, which suggests that they might share the same biosynthetic mechanisms.

Thus, mining the *S. bottropensis* ATCC 25435 genome will further elucidate the chemical and genetic diversity of this strain for the discovery of novel gene clusters and bioactive compounds. The information provided by the genome sequence is of great importance for guiding further development of bottromycin derivatives.

The genomic sequence of *S. bottropensis* ATCC 25435 not only provides a basis for the exploration of this strain for biotechnology applications but also is valuable for systematic studies of related strains.

### Nucleotide sequence accession numbers.

This Whole Genome Shotgun project has been deposited at DDBJ/EMBL/GenBank under the accession no. AOCF00000000. The version described in this paper is the first version, AOCF01000000.
